# Patients’ Knowledge, Attitudes, Behaviour and Health Care Experiences on the Prevention, Detection, Management and Control of Hypertension in Colombia: A Qualitative Study

**DOI:** 10.1371/journal.pone.0122112

**Published:** 2015-04-24

**Authors:** Helena Legido-Quigley, Paul Anthony Camacho Lopez, Dina Balabanova, Pablo Perel, Patricio Lopez-Jaramillo, Robby Nieuwlaat, J-D Schwalm, Tara McCready, Salim Yusuf, Martin McKee

**Affiliations:** 1 Department of Health Services Research and Policy, London School of Hygiene and Tropical Medicine, London, United Kingdom; 2 Fundación Oftalmológica de Santander, Bucaramanga, Colombia; 3 Instituto Masira, Medical School, Universidad de Santander, Bucaramanga, Colombia; 4 Population Health Research Institute, Hamilton, Ontario, Canada; University of Tolima, COLOMBIA

## Abstract

Hypertension is a leading cause of premature death worldwide and the most important modifiable risk factor for cardiovascular disease. Effective screening programs, communication with patients, regular monitoring, and adherence to treatment are essential to successful management but may be challenging in health systems facing resource constraints. This qualitative study explored patients’ knowledge, attitudes, behaviour and health care seeking experiences in relation to detection, treatment and control of hypertension in Colombia. We conducted in-depth interviews and focus group discussions with 26 individuals with hypertension and 4 family members in two regions. Few participants were aware of ways to prevent high blood pressure. Once diagnosed, most reported taking medication but had little information about their condition and had a poor understanding of their treatment regime. The desire for good communication and a trusting relationship with the doctor emerged as key themes in promoting adherence to medication and regular attendance at medical appointments. Barriers to accessing treatment included co-payments for medication; costs of transport to health care facilities; unavailability of drugs; and poor access to specialist care. Some patients overcame these barriers with support from social networks, family members and neighbours. However, those who lacked such support, experienced loneliness and struggled to access health care services. The health insurance scheme was frequently described as administratively confusing and those accessing the state subsidized system believed that the treatment was inferior to that provided under the compulsory contributory system. Measures that should be addressed to improve hypertension management in Colombia include better communication between health care professionals and patients, measures to improve understanding of the importance of adherence to treatment, reduction of co-payments and transport costs, and easier access to care, especially in rural areas.

## Introduction

Hypertension is one of the leading causes of premature death worldwide and remains the most important modifiable risk factor for cardiovascular and cerebrovascular disease [1, 2]. Across Latin America, the prevalence of hypertension among adults (>35 yrs) ranges from 26% to 42% [3].

Evidence from randomized trials has shown that effective treatment with blood pressure lowering medications reduces the risk of cardiovascular morbidity and mortality [4]. Nevertheless, many people remain undiagnosed, untreated, or with their blood pressure inadequately controlled even where they access the health system. The Prospective Urban and Rural Epidemiology (PURE) study, conducted in 17 countries, found that only 47% of those with hypertension were aware of it and, of those on treatment, less than a third were controlled, again with the lowest rates in poorer countries and in rural areas [5]. There are clearly many barriers to success in countries of all income levels. Progress in tackling this issue will require a comprehensive understanding of the barriers and facilitators to implementing change [6].

In two recent systematic reviews [7, 8], we have identified barriers to the control of hypertension at the service level (related to characteristics of individual providers and patient experience with frontline services) and the health system level (related to financial, organizational and governance issues). Examples of the former included difficulties with transportation; inappropriate opening hours; difficulties in making clinic appointments; inaccessible health care facilities; and lack of insurance and high costs of treatment [7]. The latter identified barriers related to health financing such as extent of insurance coverage and co-payment and delivery models such as lacking access to routine place of care or physician (Maimaris, Paty et al. 2013). However, most of the studies identified in these reviews had been undertaken in high income settings, especially the USA, with very little evidence from low or middle income country settings, and even less using qualitative methods to assess the experience of patients. Thus, the review of barriers at the service level identified four qualitative studies exploring patients’ perspectives from low-middle income countries, with only one discussing the experiences of patients in Latin America (Brazil) [7].

Policies to tackle the avoidable burden of disease associated with hypertension must be based on contextually appropriate evidence, which is currently lacking. The purpose of this paper is to begin to address this gap, by generating evidence on how patients with hypertension in one middle income country, Colombia, seek, obtain and adhere to necessary care. This is in preparation for a cluster randomised controlled trial that will evaluate a contextually appropriate intervention to improve the detection and management of hypertension in several countries.

## The Case Study Setting: Colombia

The prevalence of high blood pressure in the Colombian population aged 18–69 years old is estimated to be 23% [9], with 24.5% of patients diagnosed with hypertension receiving medication [10]. The Colombian health care system underwent a major change following the inclusion of a right to health care in a 1991 Constitutional amendment [11]. The subsequent health insurance reform (Law 100), enacted in 1993, established two schemes conferring access to a basic benefits package for large sections of the population. The mandatory mixed contributory scheme covers those who are salaried or retired, whereas the subsidized health insurance regime covers those meeting criteria for being poor. The subsidized regime is less comprehensive but covers basic health care and severe illness requiring prolonged hospitalization. Those who are not enrolled in either of these two health insurance regimes are entitled to free basic health care at public sector facilities [12].

In 2012, official records indicated that 48.9% of the population was covered by the contributory regime with 41.6% covered through the subsidized regime [13]. However, a 2008 national survey reported that 18.7% of those attending health care facilities had to bear the cost from their own resources and/or with the help of their family. Among those not seeking medical attention, the main reason was lack of financial resources (24%) [14]. Law 1438, of 2011, was designed to address inadequate coverage and fragmentation of services, seeking to remove structural barriers to care and to improve provider payment mechanisms [12], although the results have yet to be evaluated.

## Methods

### Sampling

Colombia comprises thirty-two regions. This study was undertaken in two regions, Santander and Caldas, both included in the PURE study, which gave us access to data on each community to provide context. In Santander we conducted interviews in the capital of the department Bucaramanga and suburban and rural areas, whilst in Caldas (a coffee producing region) we focused on the city Manizales and the surrounding rural areas. We chose two regions, one rural and one urban to provide a more comprehensive picture of the health system barriers faced by hypertensive patients. Interviews were undertaken in health care facilities and participants’ homes.

This study used two methods to sample key informants. The first was purposive, selecting respondents on the basis of their characteristics (age, gender, rural-urban, hypertensive status (controlled, uncontrolled) and socio-economic status). Socio-economic status was assessed according to the type of health care insurance that participants were receiving since the Colombian government provides a contributory or subsidised scheme depending on an economic assessment of the beneficiary. These participants were selected from the medical records of one of the main hospitals in Bucaramanga (FOSCAL) and in Manizales (Hospital de Caldas) and were invited to an interview by their primary health care physician and nurses. Our aim was to interview as diverse a range of individuals as possible. Second, we used a snowball sampling technique which involved asking interviewees to nominate other people they knew who may have knowledge and experience that are particularly relevant to the study. Five participants were nominated and three participants were chosen. The purpose was to include potential hypertensive patients that were not accessing the health care systems. These had to be done by asking interviewees if they knew anyone in the community that was hypertensive but was not accessing services. This allowed researchers to identify and interview patients who believed that they may have hypertension but had yet to be diagnosed.

#### Interviews and focus group discussions

This study involved semi-structured interviews and focus groups discussions with 26 individuals who either had, or suspected they might have hypertension and 4 family members. Two focus groups took place with 5 members each (6 patients and 4 family members), fourteen were interviewed individually and the remaining 6 were married couples interviewed together. Their characteristics are shown in Table 1. Interviews were conducted during August 2012 and September 2013. The average age of participants was 60 years (range 35–82 years) with 18 women and 12 men. (See Table 2 for summary of Interview Guide).

**Table 1:** Patients’ and family members’ characteristics.

Patients Characteristics	Female	Male	Total
**Age Range**			
**35–55**	2	1	3
**56–65**	2	2	4
**66–75**	11	7	18
**More than 75**	3	2	5
**Location**			
**Santander (Rural and Urban)**	15	9	24
**Caldas (Rural and Urban)**	3	3	6
**Management of Hypertension**			
**Controlled**	9	8	17
**Uncontrolled**	2	4	6
**Not clear**	3	0	3
**Family member**	4	0	4
**Type of Insurance**			
**Contributory**	4	3	7
**Beneficiary**	14	7	21
**No insurance**	0	2	2

**Table 2:** Summary Interview Guide.

**KNOWLEDGE AND DIAGNOSIS**
• Can you tell us about your first symptoms of Hypertension? Do you have other health problems?
• How did you decide to seek care? Did the family help in this process?
• Can we talk about your experience of this process?
• To what extent do you think HT is an important disease?
• How much did you know about HT before your diagnosis? What were your information sources, at the time and now?
**TREATMENT**
• What was the treatment that was first prescribed? Was it subsequently changed—in what way?
• Did you have to pay anything out-of-pocket?
• If any, what difficulties did you face during this process? [related to the health system, family, work etc.]
• Have you received any advice on preventive measures?
• What in the process of treatment could have been handled better?
**ACCESS AND USE**
• Are there shortages of drugs and consumables? Or access problems to facilities? Discuss problems.
• Can you explain to us what type of health insurance you have and what it covers?
• Have you got a particular doctor or health professional who is mainly looking after you and who knows you well?
**HEALTH CARE EXPERIENCE AND RECCOMENDATIONS**
• How would you assess your communication with health providers you have encountered?
• To what extent have you been kept informed about you treatment?
• Have you heard of any initiative to improve prevention of HT?
• From your experience what could be done to make life of people suffering from HT easier? [in prevention, in diagnosis, in treatment]
• Are there any changes that need to be made outside the health care system?

### Ethical Approval

All respondents were given an information sheet in Spanish and were asked to sign and date a consent form. If the respondent could not read the form for any reason (visually impaired etc.) the information sheet was read to them, and they signed or marked their consent. Consent was also obtained for audio-recording. All interview materials were stored securely to assure confidentiality. Respondents were able to ask questions and express their concerns. Interviewees kept a copy of both the information sheet and consent form for each participant in the study. Both the CEI-FOSCAL Colombia and LSHTM, the UK Ethics Committees approved these consent procedures.

Confidentiality was ensured by giving each participant the option of not being quoted, even anonymously, in the study and subsequent publications; and quoting participants without reference to their age, sex, professional status and role. Participants were given the option to refuse to answer any questions and/or withdraw from the study at any time. Efforts were made to conduct the interviews in a private and comfortable space that was deemed suitable for the respondent.

Ethical approval was obtained from the Comite de Etica de Investigacion CEI-FOSCAL (Protocolo HOPE-4), Fundacion Oftalmologica de Santander in Colombia and the Observational/Interventions Research Ethics Committee from the London School of Hygiene and Tropical Medicine LSHTM (ethics ref 6535).

### Analysis

We coded all interviews primarily through an inductive approach and thematic analysis, using QSR NVivo 10 Software drawing on techniques from the constant comparative method, such as line by line analysis of early interviews, naming each line and segment of data, the use of subsequent interviews to test preliminary assumptions, and discussing deviant cases [15, 16]. Analyses of family members’ responses were also included, as they were present in the focus group discussions, and reflected on some of the questions that affected them. The interviews were recorded and transcribed in full. Each excerpt includes the number of the interview and code letters (F for Female, M for male, and FG for Focus Group), setting, condition and age range, so that extracts from the same individual can be linked. In this paper, all names are pseudonyms and identifying data have been removed to maintain confidentiality.

## Results

We present our findings under five main themes identified from analysis of participants' responses. The first examines how patients found out about their condition and discusses their symptoms and knowledge of how hypertension can be prevented and controlled. The second discusses how patients respond once they knew about their condition. The third explores health system responses to their needs, focusing on access to treatment, co-payment for medicines, geographical barriers, and the role of the family in helping to overcome these barriers. The fourth discusses the relationship between the patient and the health care professional, emphasizing the attributes that shape a trusting relationship, identified as key to adherence to treatment. Finally, the fifth theme asks what patients think could be improved. These themes encapsulate responses which go from the micro level, such as providing advice on a healthy diet, to broader issues such as the need to provide equitable health care services (See Table 3 for a summary of the key themes and subthemes).

**Table 3:** Key Themes and examples of the evidence.

Themes	Examples of Evidence
**Patients’ experiences of symptoms, awareness of prevention, and knowledge and control of hypertension**	*“I felt as if I was catching the flu*, *very hot and with a high temperature*. *I live all by myself*, *so I told my neighbour I was feeling bad before going to bed*. *At seven o’clock I got up to go to the bathroom and fell down on the floor unconscious”(FG1-5*, *Urban*, *Female*, *70-80ys*, *Hypertensive)*
**Patients’ attitudes after being diagnosed: diet and exercise**	*“We go out walking before breakfast*. *We have a glass of water and we’re off*. *We leave at 7 o’clock and walk for an hour*. *I wait for my wife and we go together*, *on an empty stomach*, *and when we get back home we have breakfast*.*” (I11-12FM*, *Urban*, *Male*, *70-80ys*, *Hypertensive)*
**Health systems barriers to accessing medication**	*“The social security isn’t going to give you pills*, *there’s no money for that*, *so you’ll have to ask for charity to get the pills you need*.*” (I3F)*
**Relationship with health care**	*I*: *And you don’t trust your doctor*?
**professionals: communicating with**	*R*: *No…*
**doctors and trust**	*I*: *And why do you think this is*?
	*R*: *I don’t know*, *we started trusting the drops*, *because they were changing her medication every day and she couldn’t even walk (I8M*, *Urban*, *Male*, *70-80ys*, *Hypertensive)*.
**Things that need improving and informational need**	*“We would like access to health care to be quicker*. *Quicker and better quality medication*. *And being able to go to health care premises and ask for an appointment*. *You go to make an appointment and they give you one for the following week when one is already dying…” (FG1-5*, *Urban*, *Female*, *60-70ys*, *Hypertensive)*.

### Patients’ experiences of symptoms, awareness of prevention, and knowledge and control of hypertension

#### Patients’ experiences of symptoms and comorbidities

The diagnosis of hypertension is often precipitated by the occurrence of symptoms, which may or may not be attributable to hypertension but which prompt the patient to seek medical advice. Some are acute, such as severe headaches, dizzy spells, feeling faint, pains in an arm or the chest, or feeling unbearably hot, which they characterized “as if on fire inside”. Others are more insidious, such as feeling generally unwell, tired, or depressed or nervous, without knowing or being able to explain why they felt so badly. The following is an example of symptoms that prompted a diagnosis of hypertension. As Maria explained:

*“I felt as if I was catching the flu*, *very hot and with a high temperature*. *I live all by myself*, *so I told my neighbour I was feeling bad before going to bed*. *At seven o’clock I got up to go to the bathroom and fell down on the floor unconscious*. *An hour later I was coming round when I heard a friend calling my name outside my door*, *I told her*, *I can’t open the door*, *I’m lying on the floor dying*. *My neighbours got a little boy to climb up and look through the window*, *and he told them Doña Maria is dead*, *then everybody got together to help me*.*”*

*(FG1-5*, *Urban*, *Female*, *70-80ys*, *Hypertensive)*

A few patients had felt perfectly well, with no symptoms, but were found to have high blood pressure during routine medical checkups. For example, a patient explained:

*“My blood pressure never went up*, *it never ever went up*. *Then*, *from one moment to the next*, *the doctor told me*, *it was Dr*. *Martin*, *he told me you are suffering from high blood pressure*. *I told him I had never had high pressure*, *but if I have to take pills*, *just give them to me*, *I said*.*”*

*(I11-12FM*, *Urban*, *Female*, *60-70ys*, *Hypertensive)*

Several participants had multiple conditions and tended to focus on those, such as diabetes, other than hypertension, which they implied was less important.

#### Patients’ awareness of prevention

There was little awareness of the causes of hypertension and no-one could recall any campaigns to increase knowledge about it, although one female interviewee, formerly a voluntary worker, reported neighbourhood talks about the general prevention of illness in the past, for example advising on diet. There was a widespread view, based on information from doctors and family experience, that hypertension was hereditary. Thus, Joe speculated that hypertension may run in the family since his father and grandfather both died at a very young age from heart attacks. Questions on the role of risk factors revealed uncertainty. Marta thinks her husband’s hypertension is due to his high cholesterol and triglycerides and obesity, yet she is puzzled because she is thin and still has hypertension. One respondent, Pablo, although not then hypertensive, had a father who had died from hypertension-related illness and was advised by his doctor to adopt a low salt and fat diet and to take exercise. He reported following these instructions for three years, but as he felt well he disregarded this advice and, two years later, had developed hypertension and had to start taking medication. Some interviewees also attributed their development of hypertension to their very hard working conditions. Violence was also mentioned in a few interviews, particularly in rural areas, as an added stress in their lives which could have contributed to hypertension.

#### Patients’ knowledge of hypertension

None of the interviewees were aware that hypertension is usually symptomless and there was little awareness of the rationale for treatment. Francisco had heard that if your blood pressure rises you can have a heart attack, something that made him afraid, but he had received no information from his doctor and was uncertain about whether the risk persisted if his blood pressure was controlled. Another patient understood that hypertension could not be cured, only controlled, but he felt that this would be difficult because he believed that worries about job loss raised his blood pressure.

#### Patients’ control of hypertension medication

Most interviewees reported no problems in taking their hypertension medication, although a few described changing it, either because it was making them cough or because they perceived that it was damaging their kidneys. Some had stopped their medication once they had normal blood pressure readings as they equated this with being cured and thus having no need for prescribed medication. Overall, most interviewees reported being ‘*muy juiciosos*’; behaving responsibly and taking notice of what doctors recommend.

### Patients’ attitudes after being diagnosed: diet and exercise

Diet and exercise, as means to lose weight, play an important role in the non-pharmaceutical management of hypertension. All interviewees described eating a traditional diet, comprising pinto beans, fried food, rice, fatty meats, fried pork belly, pork scratching, corn fritters, fries food, eggs, plantains and manioc, although the variety and quantity varied according to their income. Consumption of beer and various distilled spirits, such as sugar cane liquor, was very frequent amongst the male patients, as was smoking. A relatively affluent patient described his lifestyle:

*“I used to eat huge amounts of fat*. *At least once a week I ate brains*, *kidneys*, *small intestines*, *lots of butter spread on bread*, *corn flour fritters*, *rice*, *red meat and loads and loads of butter*. *A bottle and a half of pure rum almost every night during 40 years*, *huge quantities of alcohol and plenty tobacco*.*”*

*(I23M*, *Rural*, *70-80ys*, *Hypertensive)*

Most patients diagnosed with hypertension were advised to change their diet by their doctors, and in some cases by nutritionists. There was a general recognition that a diet rich in roast meat, pork belly and other fats, sugar cane and corn distilled spirits was unhealthy. As Octavio observed:

*“It’s good to know what’s bad and what’s good for you*. *It’s usually a matter of filling your stomach when you’re hungry*, *but when you get advice about following a diet*, *you feel better and you keep to it*, *even if you don’t like it”*.

*(I25M*, *Rural*, *60-70ys*, *Hypertensive)*

All the patients reported having reduced their salt intake to a great extent or completely eliminated added salt from their diet and many thought that reducing salt was the most important means of controlling their blood pressure. Umberto reported that his wife cooked with just a little salt, no longer used meat stock cubes, and now seasoned their food with onion, garlic and paprika. Participants had greatly reduced bread, beans and fried food and were eating salads, vegetables and, especially, more fruit, such as papayas, tangerines, mangos, whilst avoiding plantains and bananas. Several patients had lost weight, which they attributed to following a healthier diet.

Nevertheless keeping to a diet was not easy. As Pedro explained

*“I just love fried pork belly*. *I hadn’t eaten any for eight months*, *but today I bought some for my wife and son and it looked so delicious that I had to eat a bit*, *and now it’s been bad for me*.*”*

*(I16M*, *Urban*, *60-70ys*, *Hypertensive)*

Most male patients had been advised to give up alcohol but found this difficult, persisting in their habit of having a couple of beers and some type of spirits with friends, although often reduced somewhat. Some patients who also had diabetes found it very difficult to reduce salt and sugar. However, in general most patients reported following a much healthier diet. Roberto explained

*“You have to make an effort to stabilize the illness*, *because the medication doesn’t cure it*, *it just stabilizes*. *So I look after myself*. *I don’t have any salt*, *I don’t eat so much fat*, *I don’t eat between meals—so really it’s up to me*, *it’s up to my willpower*.*”*

*(I26M*, *Rural*, *70-80ys*, *Hypertensive)*

Along with a diet, the other important lifestyle change for participants was greater exercise. Most participants had been advised by their doctors to walk every day and the majority reported making an effort to do so. One participant who worked from 4 a.m. until 11 p.m. daily found it impossible to do so during weekdays. Some patients with other health problems found it difficult to walk very far, but often the husband or wife motivated each other and they went for a walk together. Francisco reported:

*“We go out walking before breakfast*. *We have a glass of water and we’re off*. *We leave at 7 o’clock and walk for an hour*. *I wait for my wife and we go together*, *on an empty stomach*, *and when we get back home we have breakfast*.*”*

*(I11-12FM*, *Urban*, *Male*, *70-80ys*, *Hypertensive)*

Several interviewees reported walking for 20 minutes daily. An interviewee reported his wife’s routine:

*“She’s been going out walking round the block for the last fifteen years*. *To stop getting bored she recites the rosary while she’s walking round*. *Another thing is that she picks fifteen leaves off a tree and every time she completes once round the block she throws a leaf away*, *that’s how she knows how many times she’s been round*.*”*

*(I13-14MF*, *Urban*, *Male*, *60-70ys*, *Hypertensive)*

### Health systems barriers to accessing medication

Generic medication for hypertension is, in principle, free for all patients and many did receive free hypertension medication and found no problem in collecting it from the hospital, health centre or pharmacy

*“Yes*, *the pills are free*. *I live near the hospital and I come every month and get them straight away”*

*(I13-14FM*, *Urban*, *Female*, *60-70ys*, *Hypertensive)*.

Some of those entitled to free medication instead bought it in a pharmacy, for various reasons. They may forget to collect them on the right day every month in the health centre; they live far away and prefer to avoid the journey and paying the bus fare; or they may go to collect them, paying the necessary transport costs, and find they are not in stock in the hospital, with the only alternative being to purchase from a pharmacy, at a cost of 500 or 2000 pesos (€0.20 - €0.80). However, despite a formal entitlement, some patients did experience problems and had to pay a portion of the cost of their medication, depending on which category they have been placed by social security:

*“all these changes*, *and now we’re all badly off*, *nobody can go to the doctor because social security doesn’t cover you*, *and you haven’t got the money*, *so you can’t go to the doctor*, *I mean to say some people can’t go*.*”*

*(I22F*, *Rural*, *Female*, *60-70ys*, *Hypertensive)*

*“The social security isn’t going to give you pills*, *there’s no money for that*, *so you’ll have to ask for charity to get the pills you need*.*”*

*(I3F*, *Urban*, *Female*, *60-70ys*, *Hypertensive)*

The problem seemed especially great where the medicines were expensive. As Remedios explained:

*“A person who needs an expensive drug has a problem*, *it’s a problem*, *because she’s going to have to wait*. *When I go to collect my pills I always see people arguing*, *complaining*, *because when they get to the health centre there’s no medication*, *they tell them to come back in a fortnight*, *what I mean is these expensive drugs aren’t available*.*”*

*(I5F*, *Urban*, *Female*, *70-80ys*, *Hypertensive)*

It was not, however, clear whether the more expensive, and often branded drug was actually necessary. Thus, some patients reported that their doctor advised them to buy a more expensive medication not covered by social security. Mercedes explained,

*“The doctor said if you want to change your pill*, *buy this drug*, *and he would give you a small piece of paper*, *buy this drug because social security doesn’t give you this more expensive drug*.*”*

*(I7F*, *Urban*, *Female*, *60-70ys*, *Hypertensive)*

A few patients, mainly in rural areas, reported having paid privately for alternative medicine, described as little drops, although the doctor had advised them to stop taking them.

Some of these barriers were overcome with help from family members and neighbours. Partners normally help each other by reminding each other to take their medication; daughters help their parents by accompanying them to the doctor, tracking down the medication, and contributing to the cost of co-payments and transport costs. However, when such support was not available, interviewees recounted experiencing stress and difficulties accessing services. The following is an example of such an occurrence. Maria was living alone when she fainted. She only received help the following day when the neighbours realized something was wrong and she was taken to hospital. As she explains after this event happened:

*“And they told my son that I couldn’t live alone anymore*, *that this could happen to me again*. *Imagine*, *I have been living on my own for 10 years*, *and from that stress I started having high blood pressure*. *Because I used to eat and think*, *my god what am I doing alone inside these four walls*.*”*

*(FG1-5*, *Urban*, *Female*, *70-80s*, *Hypertensive)*

### Relationship with health care professionals: communicating with doctors and trust

The relationship with the doctor emerged as key in all the accounts of adherence to medication. In general there are two views as to how professionals are perceived by patients. Some are praised for being communicative and pleasant while others are criticised for being distant, uncaring, and not providing sufficient information. However, in most cases, whether the accounts are positive or negative, it became evident that there was an overwhelming communication problem, with interviewees often reporting that they did not share information with their doctors or ask questions when they had doubts. Part of the problem may be the limited time available for each consultation, typically only a few minutes. Another problem may be cultural in that physicians may not feel the need to explain things. Interviewees typically reported that their doctors did not explain what it means to have high blood pressure or how it can be treated.

Several accounts illustrated the lack of information about what medication is being taken and for what reasons. As Peter explained:

*“I couldn’t have the operation done*, *because they asked me what medication I was talking and I wasn’t sure… I don’t know why they don’t tell us what medication they are giving us”*.

*(I6M*, *Urban*, *Male*, *70-80ys*, *Hypertensive)*

The implicit assumption was that doctors control the information flow, telling you what to do, so the patient does not feel empowered to ask for further information. An example is provided by an interviewee who has had her medication changed but did not know why this had happened:

*R*: *At first she changed me the medication and I wasn’t feeling well*, *I was feeling dizzy*

*I*: *Do you think she changed your medication for a reason*?

*R*: *I don’t know why she changed it*, *she didn’t explain*

*(I11-12FM*, *Urban*, *Female*, *60-70ys*, *Hypertensive)*.

Thus, interviewees did not expect explanations or feel that they could ask questions. As Maria described:

*“I don’t have the sufficient trust in my doctor to tell him*, *I have pain here or there”*

*(FG1-5*, *Urban*, *Female*, *60-70ys*, *Hypertensive)*.

Interviewees also reported seeing different doctors each time, making it difficult to establish a trusting relationship. A few interviewees also reported fear of giving information to health workers. For example, Margarita mentioned that she could not follow the dietary advice provided by her nutritionist but never mentioned this to her. She could not afford to pay for the diet and she thought it was better not to tell her as she would not be able to help her anyhow

*“I never mentioned it*, *as I couldn’t afford the diet she was recommending and she can’t do anything about it*, *and I worry too much”*

*(I17F*, *Urban*, *Female*, *60-70ys*, *Hypertensive)*.

With this poor communication, a few interviewees reported accessing other type of medical treatment, such as that provided by their church since they reported having “*faith”* in the treatment provided by their priest. This was reported more often in the suburbs and rural areas:

*I*: *And you don’t trust your doctor*?

*R*: *No…*

*I*: *And why do you think this is*?

*R*: *I don’t know*, *we started trusting the drops*, *because they were changing her medication every day and she couldn’t even walk*

*(I8M*, *Urban*, *Male*, *70-80ys*, *Hypertensive)*.

### Things that need improving and informational needs

Interviewees highlighted several areas that need improvement in relation to access and effectiveness of health care provided. Most referred to the need for quicker access to health care, including timely appointments, and receiving medication of good quality and for free. There is a perception that the medication received through the non-contributory health insurance system is of lesser quality, although it was not possible to get to the root of this concern. As a participant in the focus group mentioned:

*“We would like access to health care to be quicker*. *Quicker and better quality medication*. *And being able to go to health care premises and ask for an appointment*. *You go to make an appointment and they give you one for the following week when one is already dying…”*

*(FG1-5*, *Urban*, *Female*, *60-70ys*, *Hypertensive)*.

There were calls for more easily accessible health care facilities as transport costs were very high; several interviewees also mentioned difficulties accessing specialist care. These problems were greatest in rural areas, where many people have to walk long distances to reach health care facilities. There is a desire for greater provision of information on prevention and treatment, both from health workers and in the mass media

*“Campaigns should address those that really need it*, *those who live in rural areas”*

*(I22F*, *Rural*, *Female*, *60-70ys*, *Hypertensive)*.

When participants were asked whether they thought health care was of good quality for all, the overwhelming narrative focused on inequalities in the system, with corruption mentioned as a underlying societal problem in the focus group discussions

*“our governments are so corrupt that they do whatever they want…they have a business with health care providers and they can ask for contributions… They are especially doing ugly things to pensioners*, *they are stealing money from us*, *how can it be that each pensioner has to pay 300 pesos for accessing healthcare…”*

*(FG1-5*, *Urban*, *Female*, *40-50ys*, *Family relative)*.

The story of Pedro encapsulates many of the themes identified in our results and is even more poignant as Pedro is hypertensive but has not had treatment initiated and therefore he is not receiving the required medication. His example highlights the several factors (social, systemic, structural, psychological and medical) that come into play to explain why an individual who thinks they may have high blood pressure has not been diagnosed or is receiving appropriate treatment.

Pedro is a 45 year old manual worker whose mother thinks he may be hypertensive. His mother who is also hypertensive and is controlled is worried because her son is not taking any medication. Pedro explains that he cannot access free healthcare because he has been incorrectly assigned to a higher social stratum and thus is unable to access the system for free (even though, in theory, he should be able to). The account is quite confusing, highlighting the widespread lack of information on the right to healthcare. He recounts some symptoms he experiences,

*“the symptoms I have is that I feel very hot and my left arm hurts and my head hurts a lot*, *and sometimes I jump out of bed…it is so painful*, *but I haven’t been to see the doctor”*

*(I24M*, *Urban*, *40-50ys*, *suspected Hypertensive)*.

However, he does not perceive these symptoms as important and he spends most of the interview discussing his allergies and a bump on his leg for which he cannot afford to have an operation. He mentions that he has had to spend vast amounts of money on transport costs for his mother’s medication, as she cannot attend healthcare premises by herself due to poor health. He attends doctors who prescribe alternative medicine and pays for the consultations since he reports that the only medication he ever got in a public health care facility was *paracetamol*.

He reports that sometimes his mother’s hypertension medication is not available in the public facility and he has to buy it privately. He does not believe that everybody in Colombia receives the same care. He is distrustful of doctors, saying that he would just like to be told what his medical condition is regarding his allergies and reports that doctors lie about it

*“Well I think the best solution would be that doctors tell you the truth*, *even if we have to buy the medication somewhere else*. *We can’t afford it but we can at least try to find a solution*, *I can always resort to begging for money”*

*(I24M*, *Urban*, *40-50ys*, *suspected Hypertensive)*.

The previous account highlights the numerous barriers faced by Pedro and how they impacted his access to health care. The barriers range from issues related to lack of information, a low priority given to possible hypertension in comparison to his other health problems, economic constraints in accessing medication and facilities, and distrusting the health care system and the quality of the care provided in public health care settings.

## Discussion

This qualitative study explored knowledge, behaviour and health care experiences of patients in relation to prevention and treatment of hypertension in a middle income country setting. Patients reported how their diagnosis was frequently precipitated by acute symptoms, related or unrelated to their hypertension. It is rare for anyone to have hypertension diagnosed incidentally or during preventive or outreach activities. Although the symptoms described are either non-specific or not a recognized feature of hypertension, these accounts are remarkably similar to those reported in other qualitative studies among ethnic groups and geographical regions in other low and middle income countries [17–19]. Awareness of hypertension as a distinct condition and its treatment is low. Patients that had other conditions preferred to discuss these (e.g. diabetes, allergies, surgical procedures) rather than focusing on their experience of being hypertensive. For some interviewees it was only after prompting several times that they would discuss their high blood pressure, underlining that for some it is a medical condition of *less importance*. Research by Anthony et al (2012) exploring perceptions of hypertension treatment among patients with and without diabetes in Israel found similar results. The authors described how patients expressed the view that hypertension is less important than other conditions, such as diabetes, seen as a disease that causes immediate damage whilst hypertension is considered a risk factor for future events [20].

Patients, generally, were following unhealthy diets and were unaware of any prevention strategies. Once hypertension had been diagnosed most changed their behaviour and reported doing more exercise and reducing salt consumption. Most interviewees reported taking their medication as suggested by their doctor but having had few discussions with doctors or nurses on the importance of taking them on a regular basis. However, some reported stopping the medication once they felt better and believing they were cured when their high blood pressure reading was considered normal. A study conducted with patients with hypertension who were of Caribbean origin in London (United Kingdom) found similar results although in this case the majority of participants equated normal blood pressure readings with being cured and with no need for prescribed medicine [21]. A few also expressed concern that antihypertensives damage their kidneys, a view that we also found in parallel research in Malaysia, where patients seek to counteract it by taking traditional medicines Given the consequences of irregular treatment, the importance of this finding is clear. Furthermore, a key finding of our study is that mutually trusting relationships are vital if there is to be a favourable environment where the patient and the doctor communicate effectively. One of our systematic reviews also identified poor provider-patient communications, patients' distrust in the services provided, and lack of respect for the poor as barriers to adherence to treatment[7].

Our previous research on how patients trust in a health care setting highlighted that, on the whole, patients bestow trust on those health care professionals who exhibit caring and affectionate behaviour, but also demonstrate competence in treatment. However, they are forgiving of perceived lapses in competence when other criteria were met (i.e. respect, empathy, caring attitudes) [22]. The types of trust that we have identified in the Colombian context, as reported by participants, are based on the Parsonian model of trust which is based on ‘deference’ and ‘asymmetry’ towards health care professionals [23]. The finding of this study support a proposition that a move towards trusting relationships based on reciprocity (i.e. respect/empathy) and competence (informed trust) would favour more trusting relationships and could lead to patients sharing their stories with health care professionals, therefore resulting in better adherence to treatment.

Those interviewees suspected of being hypertensive but reporting not accessing health care facilities recounted a myriad of reasons including lack of information, economic constraints, and distrusting the health care system and its quality. A few interviewees reported accessing private health care services and taking alternative medicines rather than or in conjunction with the anti-hypertensives available from public services. The health system barriers experienced in rural and urban areas were quite similar, although patients living in rural areas reported taking longer to access health care facilities (normally walking rather than taking any local transport) and relying more often on drops and alternative medicines. Pedro’s story of suspecting he was hypertensive but not accessing health care services, illustrates how access to care is situated in a complex interplay of personal, social, systemic, structural and medical factors.

Economic constraints have been pervasive in participants’ accounts and are reported as one of the main reasons for not accessing health care services. This finding is supported by one of our systematic reviews, where we found that reducing co-payments, especially for medications, was associated with improved outcomes of hypertension in multiple studies in the USA, as well as in Finland, Israel, and Brazil [8]. Therefore, reducing or abolishing copayments for anti-hypertensives would be beneficial, especially for the poorest, who reported not accessing health care facilities for fear of having to pay for their medication.

In a situation where a health system is facing a range of financial and capacity constraints, family and social networks are relied upon to step in and provide essential support through all stages of diagnosis and treatment. In Colombia, many patients rely on help provided by family members to overcome barriers. However, when the family is not providing the additional support, interviewees recounted feelings loneliness and struggles to access health services. This absence is perceived as even more problematic as their health deteriorates. For all these people, social policies and support beyond the health system need to be put in place to assist them with accessing services and promoting their wellbeing.

### Strengths and limitations of the study

The strength of this paper is that it highlights the interaction of personal, social, systemic, structural and medical factors to explain the patient’s behaviour and management of hypertension. This is one of the first qualitative studies that elicits accounts of hypertensive patients in a middle-income country in Latin America. It has also captured the views and experiences of a variety of patients. Through in-depth interviews and focus groups we have been able to elicit accounts that allow us to understand the socio-economic and health system barriers to hypertension treatment adherence. Whilst the in-depth interviews provided very rich personal information, the focus group discussion allowed for new themes to emerge that were not discussed during interviews, such as the emphasis on inequalities in access to treatment and the reported theme of corruption in the health care system and in society as a whole.

A limitation is the possibility of social desirability bias as participants might have presented their experiences and the degree of treatment adherence in a more positive light. For example, only a few participants mentioned stopping medication when they had normal blood pressure readings, whilst this has been reported as a frequent occurrence in other studies [21]. There is also some evidence that older people tend to report higher levels of satisfaction than younger people with their health care experience and therefore our reports could be more positive than that of the general population [24]. We could identify this occurrence in our interviews, where the younger generation, particularly family relatives accompanying interviewees, were more vociferous and critical of the health care system. In addition, although we made great efforts to include interviewees that lived an isolated life, and we identified one female and two males, our sampling strategy is likely to have under-selected the more marginal and least well connected patients.

However, the focus group discussions and our sampling strategy gave voice to the most vulnerable and difficult to reach populations. This is evident in reports from participants of poorer backgrounds and rural areas, who receive services of worse quality. This emphasises the importance of seeking out those in remote areas and of lower socio-economic status, who often face especially severe constraints in accessing health care but who are often excluded from social research.

## Conclusions

This study has identified several issues that should be addressed in future policies and interventions to improve hypertension management in Colombia. These include training for health professionals on the information and interaction needs of patients, with particular attention to the normally asymptomatic nature of hypertension, the importance of adherence to prescribed treatment, attention to supply of medicines to avoid shortages, improved information about entitlements to statutory coverage, reduction or, ideally, elimination of co-payments, consideration of the particular needs of patients lacking family support, and exploration of innovative solutions to reduce transport costs, especially in rural areas. Experience elsewhere shows that none of these barriers are easy to overcome, but this study highlights areas for prioritisation.
